# MRCK-Alpha and Its Effector Myosin II Regulatory Light Chain Bind ABCB4 and Regulate Its Membrane Expression

**DOI:** 10.3390/cells11040617

**Published:** 2022-02-10

**Authors:** Alix Bruneau, Jean-Louis Delaunay, Anne-Marie Durand-Schneider, Virginie Vauthier, Amel Ben Saad, Lynda Aoudjehane, Haquima El Mourabit, Romain Morichon, Thomas Falguières, Jérémie Gautheron, Chantal Housset, Tounsia Aït-Slimane

**Affiliations:** 1 Centre de Recherche Saint-Antoine (CRSA), Institute of Cardiometabolism and Nutrition (ICAN), Inserm Sorbonne Université, 75012 Paris, France; alix.bruneau@charite.de (A.B.); jean-louis.delaunay@sorbonne-universite.fr (J.-L.D.); anne-marie.durand-schneider@inserm.fr (A.-M.D.-S.); lynda.aoudjehane@inserm.fr (L.A.); haquima.el-mourabit@inserm.fr (H.E.M.); romain.morichon@sorbonne-universite.fr (R.M.); jeremie.gautheron@inserm.fr (J.G.); chantal.housset@inserm.fr (C.H.); 2 Department of Hepatology & Gastroenterology, Charité Universitätsmedizin Berlin, 13353 Berlin, Germany; 3 CNRS UMR 8104, Institut Cochin, Inserm U1016, Université de Paris, 75014 Paris, France; vauthier.v@gmail.com; 4 Inserm, Physiopathogénèse et Traitement des Maladies du Foie, UMR_S 1193, Université Paris-Saclay, Hepatinov, 91400 Orsay, France; bensaadamale@gmail.com (A.B.S.); thomas.falguieres@inserm.fr (T.F.); 5 Centre de Référence des Maladies Rares Maladies Inflammatoires des Voies Biliaires et Hépatites Auto-Immunes & Service d’Hépatologie, Assistance Publique—Hôpitaux de Paris, Hôpital Saint-Antoine, 75012 Paris, France

**Keywords:** ABC transporters, bile secretion, cholestatic liver diseases, membrane internalization

## Abstract

ABCB4, is an adenosine triphosphate-binding cassette (ABC) transporter localized at the canalicular membrane of hepatocytes, where it mediates phosphatidylcholine secretion into bile. Gene variations of ABCB4 cause different types of liver diseases, including progressive familial intrahepatic cholestasis type 3 (PFIC3). The molecular mechanisms underlying the trafficking of ABCB4 to and from the canalicular membrane are still unknown. We identified the serine/threonine kinase Myotonic dystrophy kinase-related Cdc42-binding kinase isoform α (MRCKα) as a novel partner of ABCB4. The role of MRCKα was explored, either by expression of dominant negative mutant or by gene silencing using the specific RNAi and CRISPR-cas9 strategy in cell models. The expression of a dominant-negative mutant of MRCKα and MRCKα inhibition by chelerythrine both caused a significant increase in ABCB4 steady-state expression in primary human hepatocytes and HEK-293 cells. RNA interference and CRISPR-Cas9 knockout of MRCKα also caused a significant increase in the amount of ABCB4 protein expression. We demonstrated that the effect of MRCKα was mediated by its downstream effector, the myosin II regulatory light chain (MRLC), which was shown to also bind ABCB4. Our findings provide evidence that MRCKα and MRLC bind to ABCB4 and regulate its cell surface expression.

## 1. Introduction

The ATP-binding cassette (ABC) transporter ABCB4, also called MDR3 (multidrug resistance 3), is functionally expressed in hepatocytes, where it mediates ATP-dependent translocation of the membrane phospholipid phosphatidylcholine (PC) from the inner leaflet to the outer leaflet of hepatocytes canalicular membranes (for review, see [1]). PC secreted into bile forms mixed micelles with bile acids and cholesterol, thereby preventing the formation of cholesterol gallstones and the detergent activity of non-micellar bile acids [2,3]. ABCB4 deficiency causes progressive familial intrahepatic cholestasis type 3 (PFIC3), a rare autosomal recessive disease occurring early in childhood that may be lethal in the absence of liver transplantation [4], and less-severe diseases which occur in young adults, including low-phospholipid-associated cholelithiasis (LPAC) syndrome and intrahepatic cholestasis of pregnancy (ICP) [5,6,7].

**Figure 1:**
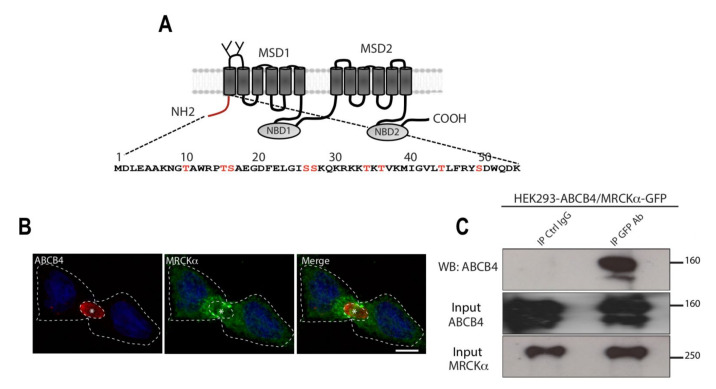
Colocalization and coimmunoprecipitation of ABCB4 with the serine/threonine kinase MRCKα. (**A**) Schematic representation of ABCB4. ABCB4 is composed of two membrane-spanning domains (MSD1 and MSD2) and two nucleotide-binding domains (NBD1 and NBD2). The two glycosylation sites in the first extracellular loop are indicated. The amino acid sequence of the intracytoplasmic N-terminal domain of human ABCB4 isoform A (NP_000434.1) is shown. The serine and threonine residues present in the N-terminal domain of ABCB4 and are indicated in red. (**B**) HepG2 cells transiently expressing ABCB4 were grown on coverslips, fixed, permeabilized, and stained with anti-ABCB4 antibody followed by anti-MRCKα antibody, and then incubated with Alexa-Fluor-594- and 488-conjugated secondary antibodies and visualized by confocal microscopy. Nuclei were stained with DRAQ 5 (Blue). Asterisks indicate bile canaliculi. Bars: 10 mm. (**C**) HEK-293 cells were co-transfected with plasmids expressing ABCB4 and GFP-tagged MRCKα, and cell lysates were incubated with anti-GFP antibody or mouse immunoglobulin G (IgG) covalently linked to agarose beads. The immunoprecipitated complex was immunoblotted with anti-ABCB4. The presence of MRCKα and ABCB4 in the cell lysate (Input) was detected by immunoblot with anti-MRCKα and anti-ABCB4 antibodies. Presented data were cropped from the full immunoblots shown in Supplementary Figure S1.

More than 500 ABCB4 variations have been identified to date, with different effects on the expression, intracellular traffic, and/or activity of ABCB4 [8,9,10]. Targeting ABCB4 to the canalicular membrane is essential for its function. However, very little is known regarding molecular partners that bind ABCB4 and specifically regulate its trafficking to and potentially from the canalicular membrane and/or PC secretion activity [11]. ABCB4 has two halves, each consisting of six membrane-spanning domains and a nucleotide-binding domain, joined by a linker region [12] (Figure 1A). HS1-associated protein X-1 (HAX-1) and myosin II regulatory light chain (MRLC) have been identified as direct binding partners of the linker domain of three ABC transporters located in the canalicular membrane of hepatocytes, i.e., the drug export pump ABCB1 (MDR1), the bile salt export pump ABCB11 (BSEP), and ABCB4. Regarding ABCB11, its apical trafficking was shown to involve MRLC, whereas clathrin-mediated endocytosis of the protein involves HAX-1 [13,14]. Whether these two molecules also play a role in the apical trafficking and potential internalization of ABCB4 is unknown. ABCB4 and ABCB1 C-terminal regions are highly conserved, with the exception of the last three amino acids. We showed that the stability and fate of ABCB4 after reaching the canalicular membrane required a carboxyl-terminal PDZ-like motif (QNL) that binds the PDZ domain protein NHERF/EBP50 [15].

The N-terminal domain of ABCB4 consists of 54 amino acids that are poorly conserved compared to those of other ABC transporters. It contains several charged amino acids and potential phosphorylation sites of serines and threonines, suggesting that it is a region of protein interaction, notably with protein kinases. We previously showed that phosphorylation of the N-terminal domain of ABCB4 regulated ABCB4-mediated PC secretion [9], although the kinases causing this phospho-regulation have not been identified yet.

We performed a yeast two-hybrid screening with the N-terminus of ABCB4 against a human liver cDNA library, and thereby identified the serine/threonine kinase Myotonic dystrophy kinase-related Cdc42-binding kinase isoform α (MRCKα), also known as Cdc42-binding protein A (Cdc42-BPA), as a binding partner of ABCB4. MRCKα protein is a serine/threonine kinase that is part of the AGC (PKA, PKG, and PKC) kinase family [16]. This kinase is a downstream effector of the GTPase-Cdc42 that plays key roles in actin-myosin dynamics (for review, see [17]). MRCKα can activate MRLC, either via direct phosphorylation [18] or via phosphorylation of its phosphatase MYPT1 [19,20].

In the present study, we demonstrate that MRCKα binds to ABCB4 in vitro. Our findings further indicate that MRCKα and its effector MRLC regulate ABCB4 cell surface expression.

## 2. Materials and Methods

### 2.1. Antibodies and Reagents

The mouse monoclonal P3II-26 anti-ABCB4 antibody was obtained from Enzo Life Sciences (Villeurbanne, France). Rabbit polyclonal anti-MRCKα and anti-MRLC were from Euromedex (Souffelweyersheim, France). Monoclonal anti-myc and anti-α-tubulin antibodies were from ThermoFisher (Cergy-Pontoise, France) and ProteinTech (Manchester, United Kingdom), respectively. The mouse monoclonal anti-GFP was from Roche (Meylan, France). Alexa Fluor-labeled secondary antibodies, DRAQ5 fluorescent probes, and culture media were from ThermoFisher (Cergy-Pontoise, France), and peroxidase-conjugated secondary antibodies were from Rockland Immunochemicals (Gilbertsville, PA). The control non-specific siRNA “ON-TARGETplus Non-targeting Control Pool”, MRCKα siRNA “ON-TARGETplus Human CDC42BPA siRNA SMARTpool”, Myl12a siRNA “ONTARGETplus Human Myl12a siRNA SMARTpool”, and Myl12b siRNA “ON-TARGETplus Human Myl12b siRNA SMARTpool” were from Dharmacon-GE Healthcare (Fontenay-sous-Bois, France). The ECL-Prime detection kit was from VWR (Courtaboeuf, France). The transfection reagents Turbofect and JetPrime were purchased from ThermoFisherScientific, (Saint-Herblain, France) and Ozyme (Saint-Cyr-l’Ecole, France), respectively. Chelerythrine chloride was obtained from Enzo Life Science (Villeurbanne, France).

### 2.2. Yeast Two-Hybrid Screen

Screening with the N-terminal domain of ABCB4 (Figure 1A) against a human liver cDNA library was performed at Hybrigenics Services per their standard protocols. The screen parameters are as follows: (1) Nature: cDNA; (2) Reference Bait Fragment: Homosapiens-ABCB4 (aa1-54); hgx3706v2; (3) Prey Library: Human Liver_RP1; (4) Vectors: pB29 (N-bait-LexA-C fusion); (5) Processed Clones: 39 (pB29_A); Analyzed Interactions: 97.4 million (pB29_A), and (6) 3AT Concentration: 0.0 mM (pB29_A).

### 2.3. DNA Constructs

The construction of the human wild type ABCB4 (ABCB4-wt) isoform A in the pcDNA3 vector has been previously described [8]. The construction of the human triple c-myc tag ABCB4-wt (3xmyc-ABCB4-wt) has been previously described [15]. The constructs of the human MRCKα, wild-type GFP-tagged MRCKα (MRCKα-wt-GFP), and kinase-dead Flag-tagged (MRCKα-KD-Flag) were provided by P. A. Gagliardi from the L. Primo laboratory (Laboratory of Cell Migration, Candiolo Cancer Institute FPO-IRCCS, 10060 Candiolo, Italy) and produced as described in [21]. The constructs of the human MRLC-wt-GFP and MRLC-AA-GFP (dominant negative mutant with threonine 18 and serine 19 mutated in alanine) were provided by Hamao Kozue from Hiroshi Hosoya laboratory (Department of Biological Science, Graduate School of Science, Hiroshima University, Higashi-Hiroshima 739-8526, Japan) and produced as described in [22]. All constructs were verified by automated sequencing.

### 2.4. Reverse Transcription Quantitative PCR (RT-qPCR)

HEK-293 cells stably expressing ABCB4-wt were transfected with MRCKα siRNA or control siRNA. After 72 h of transfection, total RNA was extracted using the RNeasy Mini Kit (Qiagen, Courtaboeuf, France). Complementary DNA was synthesized from 1 μg of total RNA using random hexamer primers and 200 U of Moloney murine leukemia virus reverse transcriptase (Life Technologies, Carlsbad, CA, USA) for 1 h at 37 °C. qPCR was performed using the Sybr Green Master Mix on a Light-Cycler 96 (Roche Diagnostics, Basel, Switzerland), with hypoxanthine phosphoribosyl-transferase as a reference gene. The primer sequences used were from Eurogentec (Angers, France): 5′-TGCGCTTCAGAGATGTTATTCT-3′ (sense) and 5′-TGCAGACAGCTTAGCTTTAGCAT-3′ (antisense).

### 2.5. CRISPR Cas9 Experiments

The pSpCas9(BB)-2A-GFP (PX458) plasmid containing the human codon optimized SpCas9 gene with 2A-EGFP and the backbone of sgRNA was used according to Feng Zhang Lab CRISPR plasmid instructions [23]. sgRNA were designed using the CRISPR Design Tool from Dharmacon, targeting exon 1 of MRCKα. The sequence of primers, Eurogentec (Angers, France), hybridized and cloned in PX458 using BbsI were MRCKα-Hs-1S 5′3′Trq (GGGCCCGCTCAGACCAAT) and MRCKα-Hs-1AS 5′3′Trq (ATTGGTCTGAGCGGGCCCC). sgRNA were designed using the CRISPR Design Tool from Dharmacon, targeting exon 2 of MYL12B. The sequence of primers, Eurogentec (Angers, France), hybridized and cloned in PX458 using BbsI were Myl12b-Hs-1S 5′3′Trq (GAGATGGCTTCATCGACA) and Myl12b-Hs-5AS 5′3′Trq (TGTCGATGAAGCCATCTCC). We used the web-based tool, CRISPOR (http://crispor.tefor.net, accessed on 20 June 2020) to ovoid off-targets effects. Constructs were verified by automated sequencing. HEK-293 cells were transfected with 3 μg of the different construction PX458-MRCKα or PX458-Myl12b with 6 μL of Turbofect (ThermoFisherScientific, Saint-Herblain, France) to generate the HEK-293 KO-MRCKα and HEK-293 KO-MRLC. To minimize the effect of possible off-target mutations, we analyzed heterogeneous populations rather than clonal populations.

### 2.6. Cell Culture, Transfection and Immunofluorescence

Human hepatocellular carcinoma HepG2 (ATCC^®^-HB-8065^TM^) cells and Human embryonic kidney HEK-293 (ATCC^®^-CRL-1573^TM^) cells were obtained from ATCC (Manassas, VA, USA). As previously reported, both HEK-293 and HepG2 cells do not express detectable endogenous ABCB4 [10]. Cells were grown at 37 °C in Dulbecco’s modified Eagles medium (DMEM), as previously reported [9]. The generation of HEK-293 cells stably expressing wild-type ABCB4 (ABCB4-wt) has been previously described [10]. Transient transfections were performed using Turbofect at a ratio of reagent:DNA of 2:1 for HEK-293 cells, and JetPrime at a ratio of reagent:DNA of 2:1 for HepG2 cells, according to manufacturer’s instructions. Immunofluorescence analyses were performed as described [10].

Primary human hepatocytes (PHHs) isolation was performed on the Human HepCell platform (ICAN, Paris, France; http://www.ican-institute.org/category/plateformes, accessed on 15 January 2019) according to the previously described protocol [24]. PHHs were treated with 20 μM of chelerythrine chloride for different time periods.

### 2.7. Coimmunoprecipitation and Western Blotting

For the coimmunoprecipitation of ABCB4 and MRCKα or MRLC, HEK-293 cells were co-transfected with plasmids encoding ABCB4 and GFP-tagged MRCKα, or GFP-tagged MRLC. Forty-eight hours after co-transfection, cells were washed with phosphate-buffered saline (PBS) and lysed at 4 °C in lysis buffer containing 25 mmol/L Tris, pH 7.4, 150 mmol/L NaCl, 1 mmol/L EDTA, 1% Triton X-100, in the presence of a protease inhibitor cocktail from Sigma-Aldrich (Lyon, France). Lysates were centrifuged at 12,000× *g* for 10 min to remove insoluble materials. Immunoprecipitation was performed overnight at 4 °C with 1 mg of protein lysate and 2 μg of anti-GFP or 2 μg of immunoglobulins from normal mouse serum pre-adsorbed onto Protein A-Sepharose beads (VWR) for 4 h at 4 °C. Immunoprecipitated proteins were subjected to immunoblotting using the monoclonal P3II-26 anti-ABCB4 antibody. Immunoblotting analyses were performed using the rabbit-polyclonal anti-MRCKα antibody or the rabbit-polyclonal anti-MRLC antibody followed by horseradish peroxidase-conjugated secondary antibodies. Immunoblotting of α-tubulin was also performed as a loading control. Development of peroxidase activity was performed with the ECL prime Western blotting detection reagent. Blot exposure times were within the linear range of detection, and signal intensities were quantified using ImageJ software.

### 2.8. siRNA Knockdown

ABCB4-wt-expressing HEK-293 cells were transfected with 75 pmol/1 mL MRCKα siRNA, 75 pmol/1 mL Myl12a siRNA, 75 pmol/1 mL Myl12b siRNA, or 75 pmol/1 mL control siRNA by incubation in the presence of JetPrime following the manufacturer’s instructions. The effect of the siRNA was analyzed 72 h after transfection, when silencing of MRCKα and MRLC were effective. Control cells were transfected with a scrambled siRNA.

### 2.9. Cell Surface Staining

HEK-293 cells-CRISPR for MRLC stably expressing 3xmyc-ABCB4-wt were transiently transfected with plasmids encoding MRLC-GFP. After 48 h of transfection, cells were washed three times with HEPES-buffered (20 mmol/L, pH 7.0) serum-free medium (HSFM). Cell surface antigens were labeled at 0 °C for 60 min with monoclonal anti-myc antibody and diluted in HSFM/0.2% BSA. After surface labeling, cells were extensively washed with HSFM/0.2% BSA and fixed, and ABCB4 was visualized with Alexa-Fluor 594-conjugated secondary antibodies. Fluorescence was examined by confocal microscopy, and the amount of ABCB4 at the plasma membrane was quantified using ImageJ software.

### 2.10. Measurement of PC Secretion

Control HEK-293 cells or HEK-CRISPR for MRLC were seeded on poly-lysine precoated six-well plates at a density of 1.3 × 10^6^ cells/well. Six hours after seeding, cells were transiently transfected with 1 μg of ABCB4-encoding plasmids using Turbofect. Twenty-four hours post-transfection, cells were washed twice with Hanks’ balance salt solution, and then the medium was replaced by phenol red-free DMEM containing 0.5 mmol/L sodium taurocholate and 0.02% fatty-acid-free bovine serum albumin (BSA) and then collected after 24 h. Measurement of PC content in the collected media was performed as described [9]. Results were normalized to the expression levels of ABCB4, which were quantified from immunoblots obtained from the corresponding cell lysates.

### 2.11. Statistical Analysis

Data were analyzed using GraphPad Prism 7.00 (La Jolla, CA, USA). Statistical analyses were performed using the Student *t* test, with a *p*-value < 0.05 considered significant, with *: *p* < 0.05; **: *p* < 0.01; ***: *p* < 0.001; ****: *p* < 0.0001; n.s.: not significant.

## 3. Results

### 3.1. MRCKα Binds the N-Terminal Domain of ABCB4

The N-terminal domain of ABCB4 is poorly conserved compared to that of other ABC transporters, suggesting that it may have a specific role in ABCB4. Moreover, this domain contains several charged amino acids and potential phosphorylation sites of serines and threonines, suggesting that it may be a region of protein interaction, in particular with protein kinases (Figure 1A). A yeast two-hybrid screen of human liver library, in which the human ABCB4 N-terminal domain was used as a bait, resulted in the identification of the serine/threonine kinase MRCKα as a new interaction partner of ABCB4. Immunofluorescence showed that in ABCB4-expressing HepG2 cells, MRCKα was localized in the cytoplasm, predominantly around the canalicular membrane (Figure 1B). To determine if, as we expected, ABCB4 forms a protein complex with MRCKα, we co-transfected HEK-293 cells with plasmids expressing ABCB4 and GFP-tagged MRCKα. The cell lysates were then incubated with anti-GFP-agarose beads, and the precipitates were analyzed by immunoblotting with anti-ABCB4. As shown in Figure 1C, ABCB4 co-precipitated together with GFP-MRCKα from co-transfected HEK-293 cells. These results demonstrated that MRCKα associates with ABCB4, suggesting that this serine/threonine kinase may regulate the expression and/or function of ABCB4.

### 3.2. MRCKα Silencing Increases ABCB4 Protein Expression

**Figure 2:**
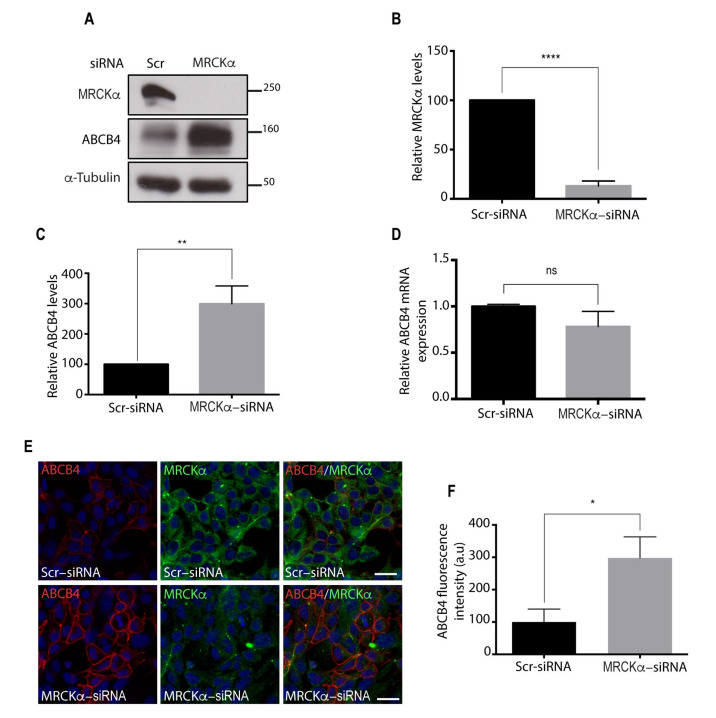
Effect of MRCKα silencing on ABCB4 protein expression. (**A**) HEK-293 cells stably expressing ABCB4 were transfected with scramble control-siRNA (Scr-siRNA) or MRCKα-siRNA. After 72 h of transfection, cells were lysed and analyzed by immunoblotting using anti-MRCKα, anti-ABCB4, and anti-a-tubulin antibodies. Presented data were cropped from the full immunoblots shown in Supplementary Figure S2. (**B**) Amounts of MRCKα were quantified from immunoblots by densitometry to assess the efficiency of the RNA interference. MRCKα levels were expressed as a percentage of total expression in HEK-293 cells transfected with Scr-siRNA. Means (±SD) of at least four independent experiments are shown. **** *p* < 0.0001. (**C**) Amounts of ABCB4 were quantified from immunoblots by densitometry. ABCB4 levels were expressed as a percentage of total expression in HEK-293 cells transfected with Scr-siRNA. Means (±SD) of at least four independent experiments are shown. ** *p* < 0.01. (**D**) RT-qPCR detected unchanged mRNA expression of ABCB4 in HEK-293 cells stably expressing ABCB4 transfected with Scr-siRNA or MRCKα-siRNA; n.s., not significant. (**E**) HEK-293 cells stably expressing ABCB4 were transfected with Scr-siRNA or MRCKα-siRNA. After 72 h of transfection, cells were fixed, permeabilized, and stained with anti-ABCB4 antibody followed by anti-MRCKα antibody, and then incubated with Alexa-Fluor-594- and 488-conjugated secondary antibodies and visualized by confocal microscopy. Nuclei were stained with DRAQ 5 (Blue). Bars: 10 mm. (**F**) The amount of ABCB4 was quantified in Scr-siRNA- or MRCKα-siRNA-transfected cells using ImageJ 1.41 Software. Means (±SD) of two independent experiments are shown. * *p* < 0.05; a.u., arbitrary units.

The role of the functional interaction of ABCB4 with MRCKα was evaluated following knock-down of MRCKα by synthetic siRNA in ABCB4-expressing HEK-293 cells. As shown by Western blot analyses (Figure 2A,B), 72 h after siRNA transfection, the level of endogenous MRCKα protein was reduced by 80% in ABCB4-expressing HEK-293 cells. The decrease in MRCKα expression caused a marked increase, up to 3-fold, in ABCB4 protein expression in HEK-293 cells transfected with MRCKα siRNA as compared to cells transfected with scramble control siRNA (Figure 2A,C). No change in ABCB4 mRNA was observed (Figure 2D), from which we inferred that MRCKα regulates ABCB4 expression via a post-transcriptional mechanism. The increase in ABCB4 protein induced by MRCKα silencing occurred at the plasma membrane, as shown by immunofluorescence (Figure 2E,F). Together, these results demonstrated that MRCKα silencing caused an increase in the amount of ABCB4 protein.

### 3.3. Inhibition of the Kinase Activity of MRCKα Increases ABCB4 Protein Expression

**Figure 3:**
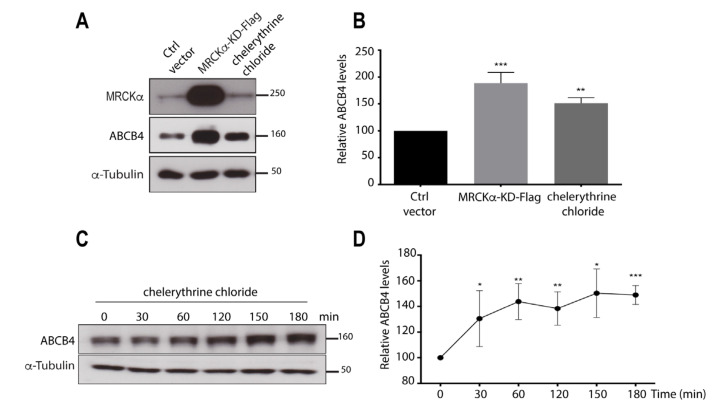
Effect of inhibition of MRCKα kinase activity on ABCB4 protein expression. (**A**) HEK-293 cells stably expressing ABCB4 were either transfected with the empty vector-Flag (ctrl vector) or Flag-tagged MRCKα-kinase-dead (MRCKα-KD-Flag) or treated with 10 mM of chelerythrine chloride for 2 h. Cells were then lysed and analyzed by immunoblotting using anti-MRCKα, anti-ABCB4, and anti-a-tubulin antibodies. (**B**) Amounts of ABCB4 were quantified from immunoblots by densitometry. ABCB4 levels were expressed as a percentage of total expression in HEK-293 cells transfected with ctrl vector. Means (±SEM) of at least eight independent experiments are shown. *** *p* < 0.001; ** *p* < 0.01. (**C**) Primary human hepatocytes were treated with 20 mM of chelerythrine chloride for the indicated time points. Cells were then lysed and analyzed by immunoblotting using anti-ABCB4 and anti-a-tubulin antibodies. Presented data were cropped from the full immunoblots shown in Supplementary Figure S3. (**D**) Amounts of ABCB4 were quantified from immunoblots by densitometry. ABCB4 levels were expressed as a percentage of total expression of untreated (Time 0 min) hepatocytes. Means (±SD) of at least three independent experiments are shown. *** *p* < 0.001; ** *p* < 0.01; * *p* < 0.05.

To determine whether the regulation of ABCB4 expression by MRCKα requires its kinase activity, we adopted a dominant-negative approach that consisted of the overexpression of a Flag-tagged MRCKα-kinase-dead (MRCKα-KD-Flag) construct. This construct was transiently transfected in ABCB4-expressing HEK-293 cells, and its effect was evaluated by Western blotting. ABCB4 protein expression was strongly increased in cells transfected with MRCKα-KD-Flag as compared to cells transfected with a control vector (Figure 3A). Quantification of Western blots showed that ABCB4 protein expression was increased to ~200% in cells transfected with MRCKα-KD-Flag (Figure 3B). In addition, we treated ABCB4-transfected HEK-293 cells with the MRCKα specific inhibitor chelerythrine chloride [17,25], which also increased the abundance of the ABCB4 protein (Figure 3A). ABCB4 protein expression was increased to 150% in treated cells, compared to untreated cells (Figure 3B). ABCB4 protein levels were also increased in freshly isolated human hepatocytes treated with chelerythrine chloride for 0–180 min (Figure 3C,D). These experiments showed that both transduced and intrinsic ABCB4 expressions are regulated by MRCKα. We inferred from these results that ABCB4 protein expression is regulated by the kinase activity of MRCKα.

### 3.4. MRCKα Knockout Increases ABCB4 Protein Expression

**Figure 4:**
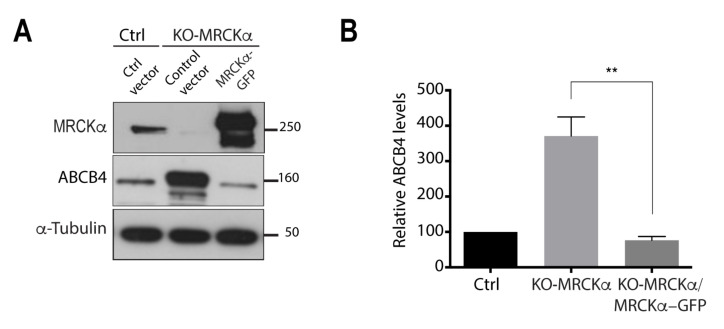
MRCKα knockout increases ABCB4 protein expression. (**A**) Control HEK-293 cells (Ctrl) or HEK-CRISPR for MRCKα (KO-MRCKα) were transfected with the empty vector-pEGFP (ctrl vector) or with MRCKα-GFP. After 24 h of transfection, they were transfected with ABCB4-wt for an additional 24 h. Cells were lysed and analyzed by immunoblotting using anti-MRCKα, anti-ABCB4, and anti-a-tubulin antibodies. Presented data were cropped from the full immunoblots shown in Supplementary Figure S4. (**B**) Amounts of ABCB4 were quantified from immunoblots by densitometry. ABCB4 levels were expressed as a percentage of total expression in Ctrl cells transfected with control vector. Means (±SD) of at least four independent experiments are shown. ** *p* < 0.01.

We generated an MRCKα knockout (KO) cell line using the CRISPR-cas9 gene editing system [23], as another approach to demonstrate the regulatory effect of MRCKα on ABCB4 protein expression. The expression of MRCKα protein was thus fully abolished in HEK-293 cells (Figure 4A). As a result, ABCB4 protein expression was significantly increased in MRCKα knockout cells (Figure 4A), with levels reaching approximately 400% of those in the controls (Figure 4B). Next, we examined if transient expression of MRCKα could rescue the MRCKα phenotype. We transfected MRCKα knockout cells with a MRCKα plasmid encoding full-length MRCKα cDNA. Immunoblots showed that transient expression of MRCKα elicited a decrease in ABCB4 expression. Quantification of Western blots showed that the expression of ABCB4 decreased from 400% to 100% and thus returned to a basal level, comparable to that observed in the control cells (Figure 4A,B). These results demonstrate that overexpression of MRCKα can rescue MRCKα knockout phenotype.

### 3.5. The Effect of MRCKα on ABCB4 Depends on Its Effector MRLC

**Figure 5:**
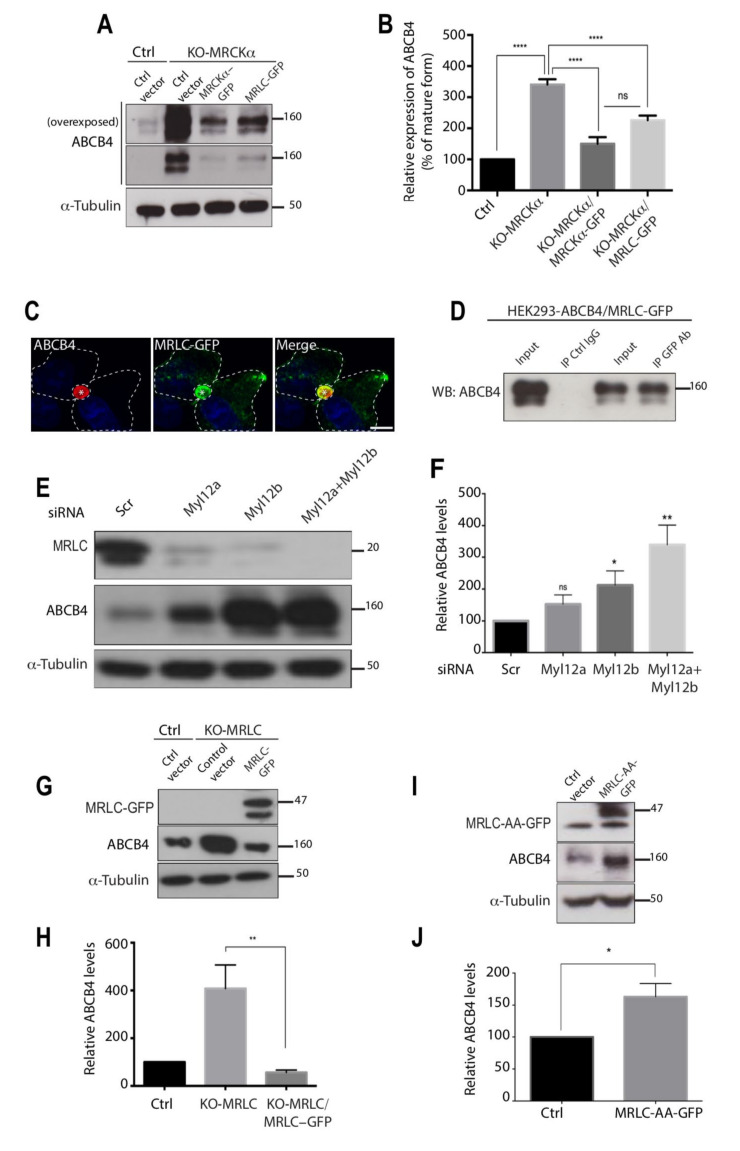
Impact of MRLC depletion on ABCB4 protein expression. (**A**) HEK-293 Ctrl or KO-MRCKα were transfected with the ctrl vector or with either MRCKα-GFP or MRLC-GFP. After 24 h, they were transfected with ABCB4-wt for an additional 24 h and analyzed by immunoblotting. The top panel shows a long immunoblot exposure for ABCB4. (**B**) ABCB4 levels were expressed as a percentage of total expression in control HEK-293 cells transfected with the control vector. Means (±SD) of at least four independent experiments are shown. **** *p* < 0.0001. (**C**) ABCB4 staining in HepG2 cells transiently expressing ABCB4 and MRLC-GFP was performed as in Figure 1B. (**D**) Cell lysates of co-transfected HEK-293 cells with plasmids expressing ABCB4 and GFP-tagged MRLC were incubated with anti-GFP antibody or mouse immunoglobulin G (IgG), covalently linked to agarose beads. The immunoprecipitated complex was immunoblotted with anti-ABCB4 antibody. The input indicates the presence of ABCB4 in the cell lysate. (**E**) HEK-293 cells stably expressing ABCB4 were transfected with control siRNA or siRNA of the two isoforms Myl12a and Myl12b of MRLC for 72 h and analyzed by immunoblotting. Presented data were cropped from the full immunoblots shown in Supplementary Figure S5. (**F**) ABCB4 levels were expressed as a percentage of total expression in HEK-293 cells transfected with the control siRNA. Means (±SD) of at least four independent experiments are shown. ** *p* < 0.01; * *p* < 0.05; n.s., not significant. (**G**) HEK-293 Ctrl or KO-MRLC were transfected with the control vector or MRLC-GFP. After 24 h, cells were transfected with ABCB4-wt for an additional 24 h and analyzed by immunoblotting. (**H**) ABCB4 levels were quantified and expressed as a percentage of total expression in the control HEK-293 cells transfected with the control vector. Means (±SD) of at least seven independent experiments are shown. ** *p* < 0.01. (**I**) HEK-293 cells stably expressing ABCB4 were transfected with the control vector or with MRLC-AA-GFP and analyzed by immunoblotting. (**J**) Amounts of ABCB4 were quantified by densitometry. ABCB4 levels were expressed as a percentage of total expression in HEK-293 cells transfected with the control vector. Means (±SD) of at least four independent experiments are shown. * *p* < 0.05.

MRLC was previously shown to be a substrate of MRCKα [19,20] and to interact with rat Mdr2, the counterpart of MDR3/ABCB4 in humans [14]. Therefore, we hypothesized that the effect of MRCKα on ABCB4 protein expression could be mediated by its effector, MRLC. To test this hypothesis, we first examined whether the overexpression of MRLC could rescue the expression level of ABCB4 comparable to the control cells in MRCKα knockout cells. MRCKα knockout cells were transfected with MRLC plasmid encoding full-length MRLC cDNA. Immunoblots showed that, much like MRCKα overexpression, the transient expression of MRLC triggered a reduction of ABCB4 expression in MRCKα knockout cells (Figure 5A,B). These results demonstrate that the overexpression of MRLC can rescue MRCKα knockout phenotype and further support the possibility that the effect of MRCKα on ABCB4 protein expression could be mediated by MRLC. We showed by immunofluorescence that ABCB4 and MRLC colocalized at the canalicular membrane in ABCB4 and MRLC-GFP-expressing HepG2 cells (Figure 5C). Next, we confirmed by immunoprecipitation that MRLC binds ABCB4 (Figure 5D). We then investigated the impact of MRLC depletion on ABCB4 protein expression. We performed an siRNA knock-down to deplete cells in one or the other MRLC isoforms, i.e., Myl12a and Myl12b, or both. As shown by Western blot analysis (Figure 5E,F), this caused a significant increase in ABCB4 protein expression, which was maximal when the two isoforms were deleted. We also generated an MRLC knockout cell line in which ABCB4 protein expression was increased to ~400% and normalized following MRLC overexpression (Figure 5G,H). These results demonstrate that the regulation of ABCB4 protein expression by MRCKα involves MRLC.

Additional experiments were performed to address the mechanism whereby MRLC regulates ABCB4 protein expression. MRLC phosphorylation at threonine 18 and serine 19 residues positively regulates myosin II activity [26], so that we postulated that MRLC phosphorylation could regulate ABCB4 protein expression. To test this hypothesis, we transfected HEK-293 cells expressing ABCB4, with plasmids expressing a non-phosphorylatable MRLC mutant in which threonine 18 and serine 19 were mutated to alanine (MRLC-AA-GFP). As a result, in the cells transfected with MRLC-AA-GFP, ABCB4 protein expression was increased to ~160% (Figure 5I,J). Although the increase in ABCB4 protein expression was moderate compared to that observed following MRLC knockout, these data suggest that MRLC phosphorylation may participate in the regulation of ABCB4 protein expression. Overall, we inferred, from these results, that ABCB4 protein expression can be regulated by the kinase MRCKα via the phosphorylation of its downstream effector, MRLC.

### 3.6. MRLC Knockout Increases ABCB4 Protein Stability and Increases Its Cell Surface Expression

**Figure 6:**
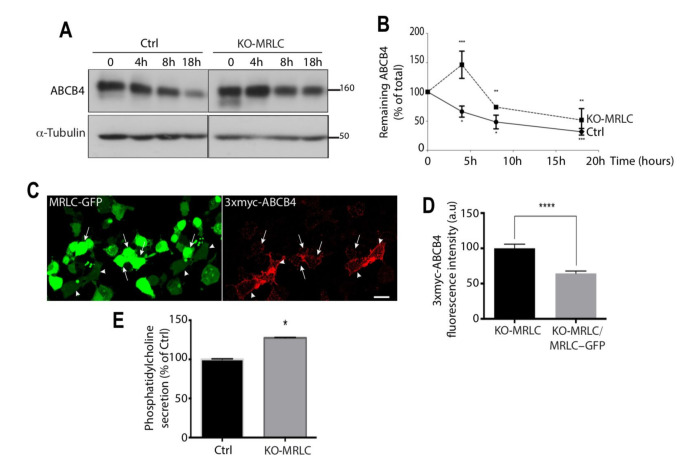
Impact of MRLC depletion on ABCB4 membrane stability. (**A**) Control HEK-293 cells (Ctrl) or HEK-CRISPR for MRLC (KO-MRLC) were transfected with ABCB4. After 24 h, cycloheximide (25 mg/mL) was added to the culture medium to inhibit protein synthesis. Expression of ABCB4 was analyzed by immunoblotting at the indicated time points, using equal amounts per line. a-Tubulin served as a loading control. Presented data were cropped from the full immunoblots shown in Supplementary Figure S6. (**B**) Amounts of ABCB4 were quantified from chase experiments. The amount of ABCB4 at time zero was considered to be 100%. The remaining ABCB4 at later time points was expressed as percentage of time zero. Means (±SD) of three independent experiments are shown. *** *p* < 0.001; ** *p* < 0.01; * *p* < 0.05. (**C**) MRLC is involved in the regulation of ABCB4 cell surface expression. HEK-293 cells-CRISPR for MRLC (KO-MRLC) stably expressing 3xmyc-ABCB4 were transiently transfected with a plasmid encoding MRLC-GFP. They were then incubated for 60 min at 0 °C with anti-myc antibody. After surface labeling, cells were fixed and ABCB4 was visualized with Alexa-Fluor 594-conjugated secondary antibody and visualized by confocal microscopy. Arrows point to MRLC knockout-3xmyc-ABCB4 expressing cells transfected with MRLC-GFP and arrowheads point to MRLC knockout-3xmyc-ABCB4 expressing cells that are not transfected with MRLC-GFP. Bars: 10 mm. (**D**) The amount of ABCB4 at the plasma membrane was quantified in MRLC knockout cells transfected with MRLC-GFP (KO-MRLC/MRLC-GFP) and compared to adjacent non-transfected cells expressing 3xmyc-ABCB4 (KO-MRLC) using ImageJ 1.41 software. Means (±SD) of at least 50 cells in two independent experiments are shown. **** *p* < 0.0001. (**E**) Impact of MRLC depletion on ABCB4 function. Control HEK-293 cells (Ctrl) or HEK-CRISPR for MRLC (KO-MRLC) were transfected with a plasmid encoding ABCB4, and PC secretion was measured after 24 h. Results are expressed as a percentage of PC secreted by ABCB4-transfected control cells with normalization to the amount of the mature ABCB4. Means (±SD) of at least two independent experiments performed in triplicate are shown. * *p* < 0.05.

To test the impact of MRLC depletion on the membrane stability of ABCB4, we analyzed the decay of ABCB4 protein expression after inhibition of protein synthesis by cycloheximide. Twenty-four hours after transfection of ABCB4 in control HEK-293 or MRLC knockout cells, 25 μg/mL cycloheximide was added to the culture medium, and the cells were harvested at specific time points for Western blot analyses (Figure 6). Figure 6A shows a representative immunoblot. At time point 0, both mature and immature forms of ABCB4 were detected in control and MRLC knockout HEK-293 cells. The immature form disappeared at later time points, consistent with inhibition of protein synthesis. Between the 4 h- and 18 h-time points, the amount of ABCB4 continuously decreased under cycloheximide treatment, both in control and MRLC knockout HEK-293 cells. However, the decay kinetics were slowed down in MRLC knockout cells compared to control cells (Figure 6A,B). These results suggested that the stability of ABCB4 was increased in MRLC knockout cells. Additional experiments were performed to determine if MRLC contributed to the internalization and recycling of ABCB4, by allowing its endocytosis. We compared the membrane staining of ABCB4 in MRLC knockout cells to that of MRLC knockout cells overexpressing MRLC. For these experiments, we used HEK-293 cells stably transfected with an ABCB4 construct bearing a triple myc-tag (3xmyc) in the first extracellular loop. This allowed specific labeling of ABCB4 localized at the plasma membrane of non-permeabilized cells [15]. MRLC knockout HEK-293-expressing 3xmyc-ABCB4 transiently transfected with MRLC-GFP plasmids encoding full-length MRLC cDNA fused to GFP were incubated with anti-myc antibodies at 0 °C to allow binding of the antibody to the ABCB4 molecules expressed at the cells surface. After 60 min, the cells were fixed and incubated with fluorescently labeled secondary antibodies. Confocal microscopy analysis showed that some cells were transfected with MRLC-GFP, but not all, thus making it possible to compare the plasma membrane staining of ABCB4 in both MRLC knockout HEK-293-expressing 3xmyc-ABCB4 and MRLC knockout HEK-293-expressing 3xmyc-ABCB4 overexpressing MRLC-GFP within the same field. We found that the plasma membrane expression of ABCB4 was reduced in MRLC knockout cells overexpressing MRLC (Figure 6C). Quantification of the fluorescence of ABCB4 at the plasma membrane showed that the intensity was reduced by 40% in MRLC knockout cells overexpressing MRLC (Figure 6D). Overall, these data provide evidence that MRLC regulates the membrane expression of ABCB4 and might be involved in its internalization from the plasma membrane.

### 3.7. MRLC Knockout Increases ABCB4 Function

We next examined if MRLC knockout cells in which the amount of ABCB4 protein was increased also showed an increase in PC secretion by ABCB4. PC secretion activity of ABCB4 was measured in the culture medium of control and MRLC knockout HEK-293 cells after transient transfection, as described [9]. The amount of PC released over 24 h was normalized for the level of the mature form of ABCB4 expressed in the corresponding cell culture condition. Figure 6E shows that the PC secretion activity of ABCB4 was significantly increased in MRLC knockout HEK-293 cells, as compared to control HEK-293 cells. We inferred from these results that ABCB4 function can be regulated by MRLC.

## 4. Discussion

It is well established that the phospholipid transporter ABCB4 needs to be at the canalicular membrane to exert its function. The molecular mechanisms that control the amount of ABCB4 protein at the cell surface have not been identified yet. We previously reported that the stability of ABCB4 at the canalicular membrane required the interaction of its C-terminal PDZ-like motif with the scaffold protein EBP50 [15], and that the phosphorylation of its N-terminal domain regulated its PC secretory function [9]. The N-terminal domain of ABCB4 contains several serines and threonines that are potential targets of protein kinases. In the present study, we identified the serine/threonine kinase MRCKα and its downstream effector MRLC as binding partners of ABCB4. By modulating the expression of MRCKα and MRLC, we demonstrated an essential role of these new partners in the regulation of ABCB4 expression at the cell surface.

We also investigated the mechanism by which MRCKα and MRLC regulate ABCB4 expression at the plasma membrane. MRLC is a substrate of MRCKα [19,20]. Therefore, we hypothesized that activation of MRLC by MRCKα could regulate the ABCB4 expression at the cell surface. Consistent with this hypothesis, the downregulation of MRLC caused an increase in the amount of ABCB4 protein. The expression of a dominant negative MRLC in which the threonine 18 and serine 19 were replaced by non-phosphorylatable alanine also caused an increase in ABCB4 protein expression. Our results suggest that MRLC controls ABCB4 protein expression negatively by stimulating its retrieval from the plasma membrane. Strong evidence supporting this hypothesis was provided by the reversal of ABCB4 membrane accumulation after overexpression of MRLC in MRLC knockout cells (Figure 6C,D). An increase in ABCB4-mediated phosphatidylcholine secretion occurred as a result of ABCB4 membrane accumulation in MRLC knockout cells. In keeping with our data, Bajaj et al. demonstrated that the inhibition of MRLC phosphorylation prevented its interaction with ABCB1, the multidrug export pump (MDR1), which was responsible for an increase in ABCB1 activity [27]. An interaction of MRLC with ABCB11, the bile salt export pump (BSEP), has also been reported, but in this case, the interaction was shown to be required for the trafficking of ABCB11/BSEP to the apical surface [14]. Thus, the expression of a dominant negative, non-phosphorylatable MRLC mutant severely impaired the delivery of newly synthesized ABCB11/BSEP to the apical surface of polarized Madin–Darby canine kidney (MDCK) cells. This illustrates the different molecular mechanisms of ABCB4 and ABCB11 internalization. ABCB11 possesses a tyrosine motif in its cytoplasmic tail, which interacts with the adaptor protein AP2, allowing its internalization and recycling [28,29]. ABCB4 does not contain such a motif, consistent with a distinct mechanism of internalization.

The recent description of PFIC6 highlights the role of the myosin family in the regulation of bile secretion and the pathogenesis of hereditary cholestatic diseases. Variations in the MYO5B gene have been identified in patients with a PFIC-like phenotype but no mutations in any of the canalicular transporters classically involved in PFICs [30]. MYO5B is an essential protein for the recycling of ABCB11 and ABCC2, the canalicular transporter of bilirubin, from Rab8 and Rab11 positive compartments. When MYO5B is mutated or truncated in vitro, ABCC2 displays an intracellular localization in Rab8 and Rab11 positive compartments. Therefore, a defect in the recycling of canalicular transporters is likely responsible for the development of PFIC6 [31].

**Figure 7:**
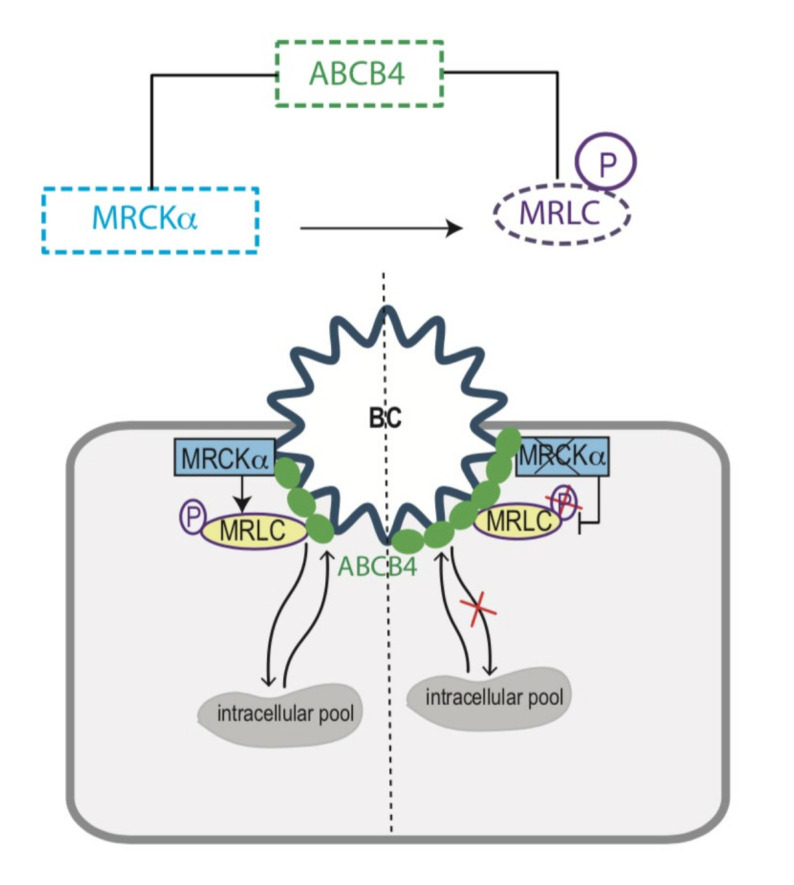
A model for ABCB4 canalicular membrane expression regulation. In the presence of MRCKα and phosphorylated MRLC, ABCB4 is internalized from the canalicular membrane. In MRCKα knockout cells, MRLC is not phosphorylated, resulting in an accumulation of ABCB4 at the canalicular membrane.

Other studies have demonstrated the involvement of the ROCK/MRLC pathway in acquired cholestasis, such as in drug-induced liver injury (DILI). Sharanek et al. showed that a dozen of the components responsible for DILI could be divided in two categories: (i) those activating the ROCK kinase, which triggered MRLC phosphorylation and bile canaliculi contraction, and (ii) those inhibiting ROCK, which prevented MRLC phosphorylation and resulted in a dilatation of bile canaliculi [32,33]. The two kinases MRCKα and ROCK have common activators and effectors, so that the MRCKα/MRLC and ROCK/MRLC pathways may act alike in the progression of cholestasis. Our functional studies suggest that MRCKα activates MRLC, which binds ABCB4 and allows its internalization from the canalicular membrane (Figure 7). In agreement with this view, Cantore et al. reported that the Src family kinase Fyn induced ABCC2 and ABCB11 retrieval from the canalicular membrane, probably by increasing cortactin phosphorylation [34]. In HUH-NTCP cells, Schonhoff et al. observed that taurolithocholate-induced ABCC2 retrieval from the membrane involved the phosphorylation by protein kinase Cε of a membrane-bound F-actin crosslinking protein, Myristoylated Alanine-Rich C-Kinase Substrate (MARCKS). They showed that in HUH-NTCP, cells transfected with phosphorylation deficient MARCKS, taurolithocholate, failed to decrease ABCC2 at the plasma membrane [35]. More recently, Wenzel et al. identified MARCKS, also as a key factor for the membrane expression of ABCB1. They showed that functional disruption of MARCKS led to an inhibition of ABCB1 internalization, resulting in its accumulation at the plasma membrane [36]. In another study, Chai et al. showed that the activation of liver PKCs led to Ezrin Thr567 phosphorylation, resulting in ABCC2 internalization [37].

In summary, our findings indicate that ABCB4 cell surface expression is mediated by the functional interaction between ABCB4, the kinase MRCKα, and its downstream effector MRLC. ABCB4 stability at the canalicular membrane is mediated by the C-terminal QNL motif, which constitutes a canalicular membrane retention motif via its interaction with the PDZ protein EBP50 [15]. These different mechanisms need to be finely coordinated to ensure normal bile secretion. In conclusion, the results presented in this work pave the way for future investigations on the molecular mechanisms underlying the canalicular membrane localization of ABCB4, which will guide the development of new therapeutic strategies for patients with liver diseases related to ABCB4 defects.

## Data Availability

Not applicable.

## Supplementary Materials

The following supporting information can be downloaded at: https://www.mdpi.com/article/10.3390/cells11040617/s1, Figure S1: Full immunoblots related to Figure 1C. Results shown in Figure 1C are delineated by dotted rectangles. MW (in kDa) are indicated. Figure S2: Full immunoblots related to Figure 2A. Results shown in Figure 2A are delineated by dotted rectangles. MW (in kDa) are indicated. Figure S3: Full immunoblots related to Figure 3A,C. Results shown in Figure 3A,C are delineated by dotted rectangles. MW (in kDa) are indicated. Figure S4: Full immunoblots related to Figure 4A. Results shown in Figure 4A are delineated by dotted rectangles. MW (in kDa) are indicated. Figure S5: Full immunoblots related to Figure 5A,D,E,G,I. Results shown in Figure 5A,D,E,G,I are delineated by dotted rectangles. MW (in kDa) are indicated. Figure S6: Full immunoblots related to Figure 6A. Results shown in Figure 6A are delineated by dotted rectangles. MW (in kDa) are indicated.

## Abbreviations

ABC	ATP-Binding Cassette
HEK	Human Embryonic Kidney
HS1	Hematopoietic cell specific protein 1
KO	KnockOut
MRCK	Myotonic dystrophy kinase-Related Cdc42-binding Kinase
MRLC	MyosinII Regulatory Light Chain
PC	PhosphatidylCholine
siRNA	small interfering RNA
WT	Wild Type
