# Supplementary material for: MRCK-Alpha and Its Effector Myosin II Regulatory Light Chain Bind ABCB4 and Regulate Its Membrane Expression
Source: Cells. 2022 Feb 10;11(4):617. doi: 10.3390/cells11040617 (PMC8870398; doi:10.3390/cells11040617)
Supplement: Supplementary file 1 [file cells-11-00617-s001.zip › cells-1535928-supplementary.pdf]

MRCK-alpha and its effector myosin II regulatory light chain bind ABCB4 and regulate its membrane expression

Alix Bruneau[1,2], Jean-Louis Delaunay[1], Anne-Marie Durand-Schneider[1], Virginie Vauthier[3], Amel Ben Saad[4], Lynda Aoudjehane[1], Haquima El Mourabit[1], Romain Morichon[1], Thomas Falguières[4], Jérémie Gautheron[1], Chantal Housset[1,5], and Tounsia Aït-Slimane[1]*

[1]Sorbonne Université, Inserm, Centre de Recherche Saint-Antoine (CRSA), Institute of Cardiometabolism and Nutrition (ICAN), F-75012 Paris, France.

[2] Department of Hepatology & Gastroenterology, Charité Universitätsmedizin Berlin, 13353 Berlin, Germany

[3]Université de Paris, Institut Cochin, Inserm U1016, CNRS UMR 8104, 75014 Paris, France

[4] Inserm, Physiopathogénèse et traitement des maladies du foie, UMR_S 1193, Université Paris-Saclay, Hepatinov, 91400 Orsay, France.

[5]Assistance Publique - Hôpitaux de Paris, Hôpital Saint-Antoine, Centre de Référence des Maladies Rares - Maladies Inflammatoires des Voies Biliaires et Hépatites auto-immunes & Service d'Hépatologie, F-75012 Paris, France.

[*] **Corresponding author :** Tounsia Aït-Slimane, PhD—Sorbonne Université, Inserm, Centre de Recherche Saint-Antoine (CRSA) — 27 Rue Chaligny—75012 Paris, France. Phone : +33 (0)1-40-01-13-56. E-mail : tounsia.ait-slimane@inserm.fr

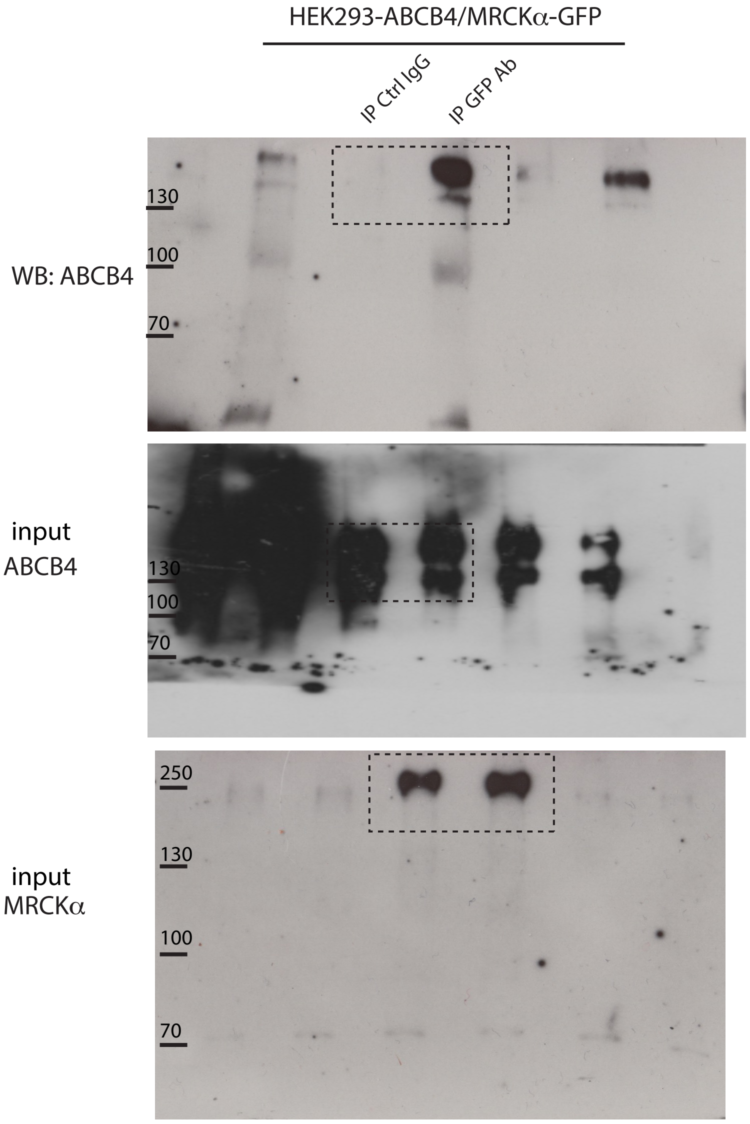

Figure S1. Full immunoblots related to Figure 1C. These immunoblots are representative of three independent experiments. Results shown in Figure 1C are delineated by dotted rectangles. NM (in kDa) are indicated

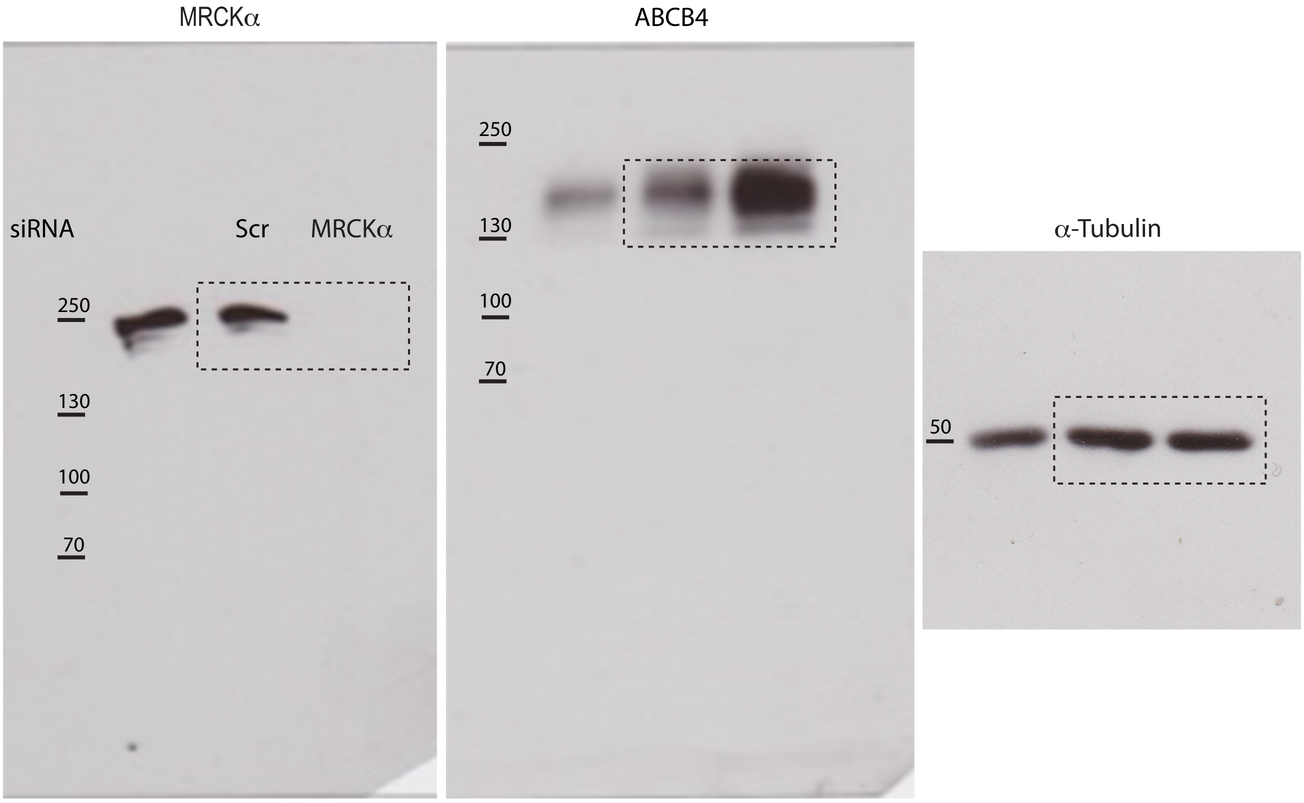

Figure S2. Full immunoblots related to Figure 2A. These immunoblots are representative of four independent experiments. Results shown in Figure 2A are delineated by dotted rectangles. NM (in kDa) are indicated

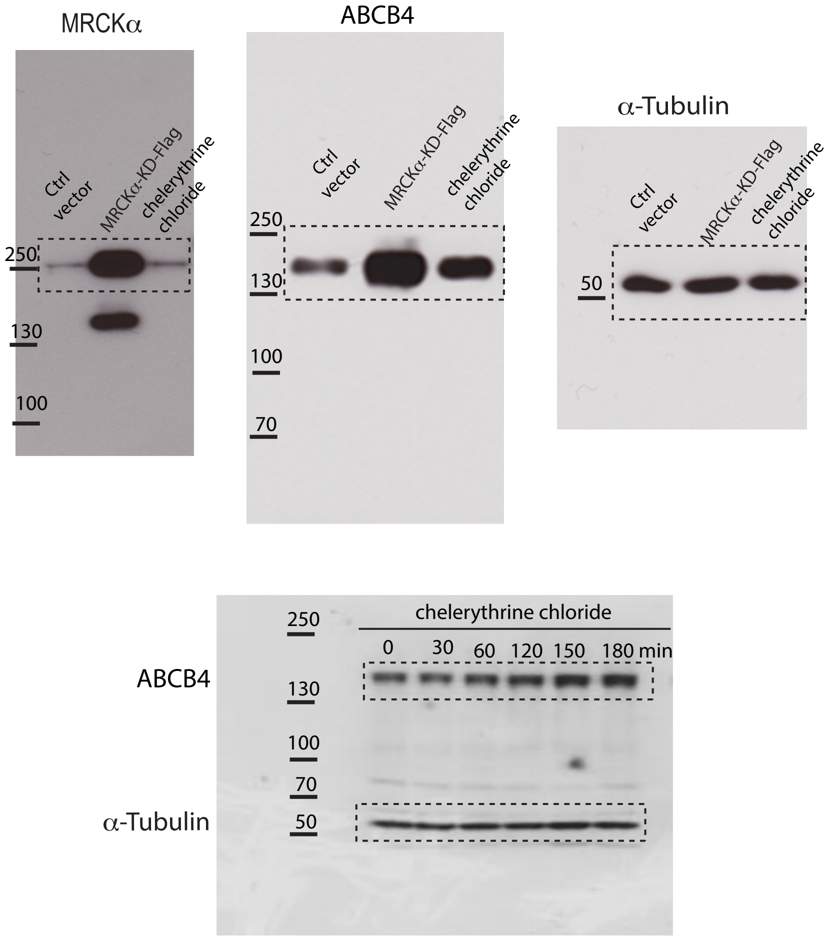

Figure S3. Full immunoblots related to Figure 3A,C. These immunoblots are representative of eight (3A) and three (3C) independent experiments. Results shown in Figure 3A,C are delineated by dotted rectangles. NM (in kDa) are indicated

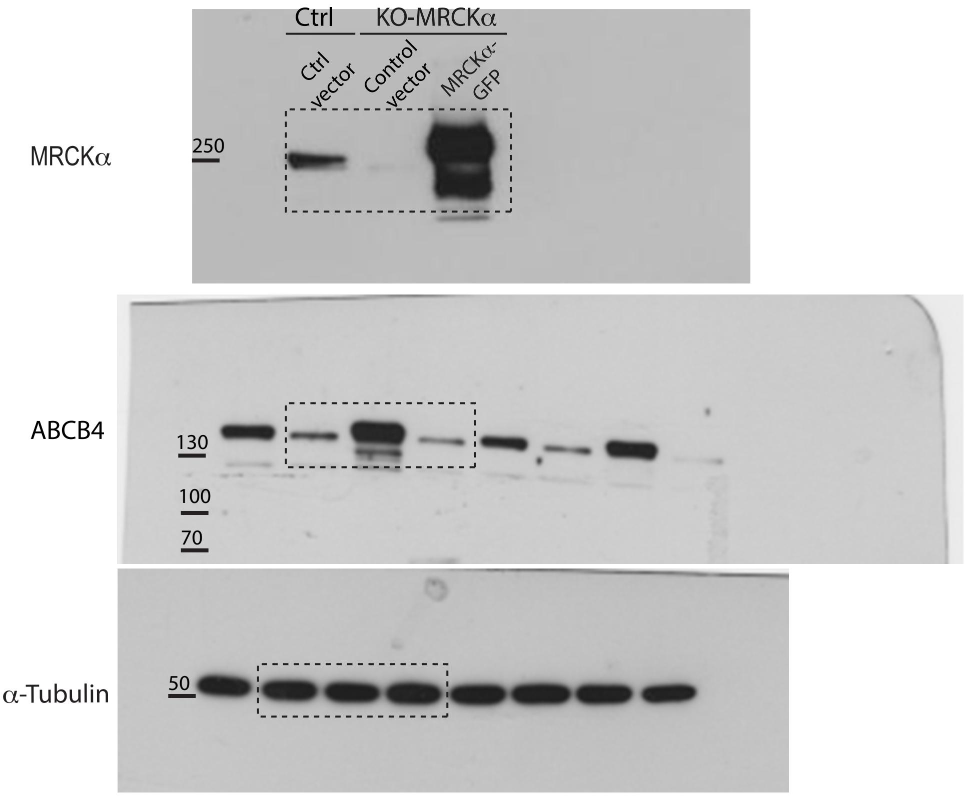

Figure S4. Full immunoblots related to Figure 4A. These immunoblots are representative of four independent experiments. Results shown in Figure 4A are delineated by dotted rectangles. NM (in kDa) are indicated

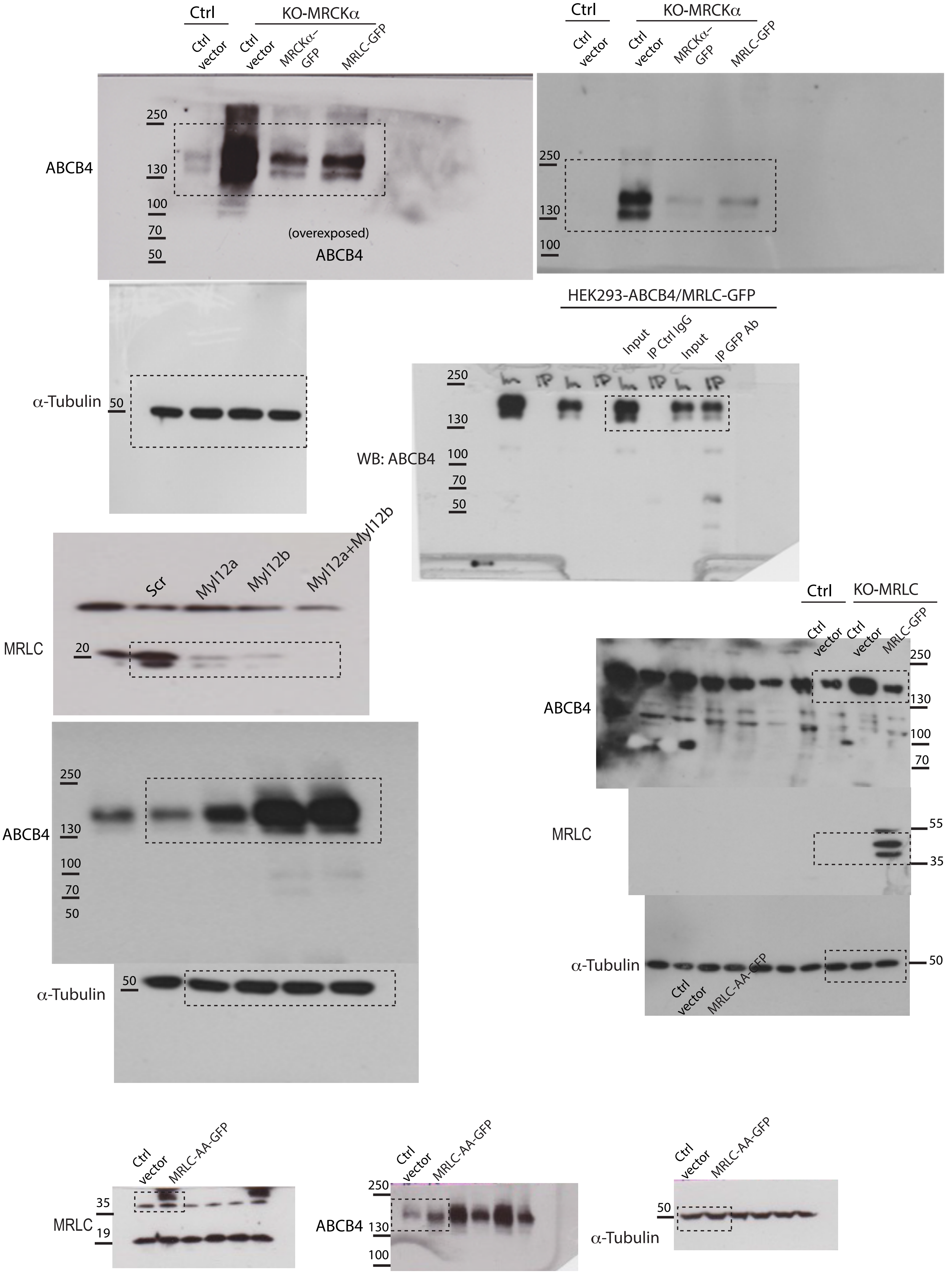

Figure S5. Full immunoblots related to Figure 5A,D and E. These immunoblots are representative of four (5A), three (5D), four (5E), seven (5G) and four (5H) independent experiments. Results shown in Figure 5A,D, E,G and H are delineated by dotted rectangles. NM (in kDa) are indicated

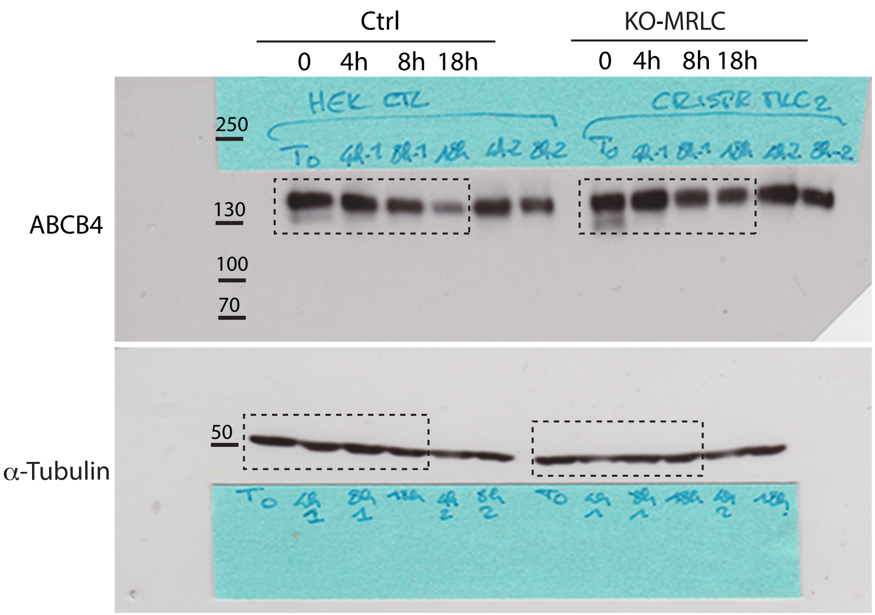

Figure S6. Full immunoblots related to Figure 6A. These immunoblots are representative of three independent experiments. Results shown in Figure 6A are delineated by dotted rectangles. NM (in kDa) are indicated
